# Relationship between the Biofilm-Forming Capacity and Antimicrobial Resistance in Clinical *Acinetobacter baumannii* Isolates: Results from a Laboratory-Based In Vitro Study

**DOI:** 10.3390/microorganisms9112384

**Published:** 2021-11-18

**Authors:** Matthew Gavino Donadu, Vittorio Mazzarello, Piero Cappuccinelli, Stefania Zanetti, Melinda Madléna, Ádám László Nagy, Anette Stájer, Katalin Burián, Márió Gajdács

**Affiliations:** 1 Hospital Pharmacy, Azienda Ospedaliero Universitaria di Sassari, 07100 Sassari, Italy; 2 Department of Biomedical Sciences, University of Sassari, Sassari, 07100 Sassari, Italy; vmazza@uniss.it (V.M.); pcappuc@gmail.com (P.C.); zanettis@uniss.it (S.Z.); 3 Department of Oral Biology and Experimental Dental Research, Faculty of Dentistry, University of Szeged, Tisza Lajos körút 63, 6720 Szeged, Hungary; madlenamelinda784@gmail.com (M.M.); mariopharma92@gmail.com (M.G.); 4 Department of Prosthodontics, Faculty of Dentistry, University of Szeged, Tisza Lajos körút 62-64, 6720 Szeged, Hungary; nagy.adam.laszlo@stoma.szote.u-szeged.hu; 5 Department of Periodontology, Faculty of Dentistry, University of Szeged, Tisza Lajos körút 62-64, 6720 Szeged, Hungary; stajeranette@gmail.com; 6 Department of Medical Microbiology, Albert Szent-Györgyi Health Center and Faculty of Medicine, University of Szeged, Semmelweis Utca 6, 6725 Szeged, Hungary; burian.katalin@med.u-szeged.hu; 7 Faculty of Medicine, Institute of Medical Microbiology, Semmelweis University, Nagyvárad tér 4, 1089 Budapest, Hungary

**Keywords:** *Acinetobacter baumannii*, antibiotic resistance, multidrug resistance, MDR, biofilm, crystal violet, phenotypic assay

## Abstract

The relationship between the multidrug-resistant (MDR) phenotype and biofilm-forming capacity has been a topic of extensive interest among biomedical scientists, as these two factors may have significant influence on the outcomes of infections. The aim of the present study was to establish a possible relationship between biofilm-forming capacity and the antibiotic-resistant phenotype in clinical *Acinetobacter baumannii* (*A. baumannii*) isolates. A total of n = 309 isolates were included in this study. Antimicrobial susceptibility testing and the phenotypic detection of resistance determinants were carried out. The capacity of isolates to produce biofilms was assessed using a crystal violet microtiter-plate-based method. Resistance rates were highest for ciprofloxacin (71.19%; n = 220), levofloxacin (n = 68.61%; n = 212), and trimethoprim-sulfamethoxazole (n = 66.02%; n = 209); 42.72% (n = 132) of isolates were classified as MDR; 22.65% (n = 70) of tested isolates were positive in the modified Hodge-test; the overexpression of efflux pumps had significant effects on the susceptibilities of meropenem, gentamicin, and ciprofloxacin in 14.24% (n = 44), 6.05% (n = 19), and 27.51% (n = 85), respectively; 9.39% (n = 29), 12.29% (n = 38), 22.97% (n = 71), and 55.35% (n = 170) of isolates were non-biofilm-producing and weak, moderate, and strong biofilm producers, respectively. A numerical, but statistically not significant, difference was identified between the MDR and non-MDR isolates regarding their biofilm-forming capacity (MDR: 0.495 ± 0.309 vs. non-MDR: 0.545 ± 0.283; *p* = 0.072), and no association was seen between resistance to individual antibiotics and biofilm formation. Based on numerical trends, MER-resistant isolates were the strongest biofilm producers (*p* = 0.067). Our study emphasizes the need for additional experiments to assess the role biofilms have in the pathogenesis of *A. baumannii* infections.

## 1. Introduction

Due to the growing number of patients affected by invasive medical interventions and immunosuppression, the prevalence and disease burden of infections caused by nonfermenting Gram-negative bacteria (NGFNBs; including *Pseudomonas aeruginosa*, *Acinetobacter* spp., and *Stenotrophomonas maltophilia*) have also increased considerably [1]. The genus *Acinetobacter* is an oxidase-negative coccobacillus, consisting of >40 genospecies, among which the *Acinetobacter baumannii-calcoaceticus complex* is the most clinically relevant [2]. *A. baumannii* is an important opportunistic pathogen in nosocomial infections, which—although traditionally considered a low-grade pathogen—has been implicated in a wide array of infectious syndromes, including bacteremia, pneumonia (especially ventilator-associated pneumonia (VAP)), urinary tract infections, and eye and wound infections, affecting patients with immunosuppression, severe underlying conditions, or patients in critical condition (treated in Intensive Care Units (ICUs)) [3,4,5,6]. Patients experiencing prolonged (≥90 days) hospitalization and previous antimicrobial therapy are particularly at risk [7]. The reported mortality rate of invasive *A. baumannii* infections is considerably high, ranging between 23% and 68% and between 0% and 64% for nosocomial and community-associated infections, respectively [8]. In a recent meta-analysis of 114 studies, the estimated mortality of *A. baumannii* VAP has been reported at 42.6% (95% CI, 37.2–48.1%), while other studies have shown that the mortality rate of the *A. baumannii* bacteremia in ICUs was between 37% and 52%, while for VAP, this figure might be as high as 84% [9].

Since the 2000s, the emergence of multidrug-resistant (MDR) and extensively drug-resistant (XDR) strains of *A. baumannii* has become a critical concern for healthcare professionals worldwide, with an alarmingly low number of antibiotics left useful for their treatment [10]. The previously mentioned meta-analysis reported the prevalence of MDR *A. baumannii* in nosocomial pneumonia at 79.9% (95% CI, 73.9–85.4%) in some regions (Central America, Latin America, and the Caribbean), where all isolates are now considered MDR [9]. In addition to their nonsusceptibility to many commonly used antimicrobials, *Acinetobacter* spp. are characterized by a considerable genomic plasticity, and a tremendous capacity to uptake novel resistance determinants (via plasmids, transposons, and integrons, accumulating them in large genomic islands in their chromosome), leading to infections where they are no longer a safe and effective drug for treatment [11]. Carbapenem-resistant *A. baumannii* (CR-AB) has been designated as a “*critical priority*” pathogen for the development of novel antibiotics and anti-infectives, according to the report published by the World Health Organization (WHO) [12].

Biofilms are complex assemblages of bacteria and an external matrix material, consisting of exopolysaccharide (EPS) and other carbohydrates, proteins, lipids, environmental DNA (eDNA), ions, and water [13,14]. Biofilms allow for a protective form of growth and metabolic inactivity for microorganisms, providing a safe haven against environmental stressors (sheer forces, heat and drying damage), attacks of the immune system (phagocytes, complement), disinfectants, and antimicrobials [15]. In fact, bacteria embedded in deeper layers of the biofilm seldom come into contact with antibiotics, due to the inability of these drugs to adequately penetrate into its deeper layers; this results in 10–1000-fold higher minimum inhibitory concentration (MIC) values, compared to the ones observed for planktonic cells [16]. The biofilm-forming capacity of *A. baumannii* largely contributes to its success as a nosocomial pathogen, as it allows for survival within hospital environments (e.g., taps and fluid containers) and on vascular catheters or other implantable devices [17]. It has been described that *A. baumannii* may survive for 4–5 months on abiotic surfaces, while so-called “hypervirulent” forms of *A. baumannii* were also associated with strong biofilm production [16,17]. The relationship between the antimicrobial-resistant phenotype and the biofilm-forming capacity has been a topic of extensive interest among biomedical scientists, as these two factors may have significant influence on the outcomes of infections [18,19]. In addition, there have been studies showing that nonlethal concentrations of antibiotics induced biofilm production in various Gram-positive and Gram-negative bacteria, suggesting a common response to overcome external stressors [20]. The aim of the present study was to establish a possible relationship between biofilm-forming capacity and the antibiotic-resistant phenotype in clinical *A. baumannii* isolates.

## 2. Materials and Methods

### 2.1. Collection of Isolates

A total of three hundred and nine (n = 309) *A. baumannii* isolates were included in this study, which were kindly provided from the strain collections of a Hungarian and Italian tertiary-care hospital. The study used a cross-sectional design, with microorganisms that were isolated between 1 January 2019 and 1 January 2020, originating from various types of clinical specimens, being randomly selected to be included in our experiments. During the study, *A. baumannii* clinical isolate no. 59738 (MDR isolate, weak biofilm producer) and *A. baumannii* ATCC 19606 (susceptible isolate, strong biofilm producer) were used as control strains (the latter was obtained from the American Type Culture Collection, Manassas, VI, USA) [21]. Stock cultures were stored at −80 °C in a cryopreservation medium (700 µL of trypticase soy broth + 300 µL of 50% glycerol) until use.

### 2.2. Re-Identification of Isolates

All isolates included in our study were re-identified as *A. baumannii* before further experiments. Re-identification of isolates was carried out using matrix-assisted laser desorption/ionization–time-of-flight mass spectrometry (MALDI–TOF MS; Bruker Daltonics, Bremen, Germany); to perform the MALDI–TOF assay, bacterial cells from fresh overnight cultures were transferred to a stainless-steel target. An on-target extraction was performed by adding 1 µL of 70% formic acid prior to the matrix. After drying at room temperature, the cells were covered with 1 µL of matrix (α-cyano-4-hydroxy cinnamic acid in 50% acetonitrile/2.5% trifluoro-acetic acid; Bruker Daltonics, Bremen, Germany). Mass spectrometry analyses were performed by a MicroFlex MALDI Biotyper (Bruker Daltonics, Bremen, Germany) in positive linear mode across the m/z range from 2 to 20 kDa; for each spectrum, 240 laser shots at 60 Hz in groups of 40 shots per sampling area were collected [22]. For analyses of spectra, the MALDI Biotyper RTC 3.1 software and the MALDI Biotyper Library 3.1 (Bruker Daltonics. Bremen, Germany) were utilized. After analysis, a log(score) value was assigned to all isolates, indicating the reliability of MALDI–TOF MS identification. The log(score) values were evaluated as follows: a log(score) <1.69 showed unreliable identification, 1.70–1.99 corresponded to probable genus-level identification, 2.00–2.29 corresponded to reliable genus-level identification, while a score ≥2.30 corresponded to reliable species-level identification [23].

### 2.3. Antimicrobial Susceptibility Testing and Resistotyping

Antimicrobial susceptibility testing for respective isolates was carried out using the Kirby–Bauer disk diffusion method (Oxoid, Basingstoke, UK), and in subsequent experiments (when relevant) with E-tests (Liofilchem, Roseto degli Abruzzi, Italy) on Mueller-Hinton agar (MHA) (bioMérieux, Marcy-l’Étoile, France) plates in the case of imipenem (IMI; 10 µg), meropenem (MER; 10 µg), ciprofloxacin (CIP; 5 µg), levofloxacin (LEV; 5 µg), trimethoprim-sulfamethoxazole (SXT; 23.75/1.25 µg), gentamicin (GEN; 10 µg), and amikacin (AMI; 30 µg). Tigecycline (TIG) susceptibility was performed using E-tests on MHA, while colistin (COL) susceptibility was performed using the broth microdilution method in cation-adjusted Mueller-Hinton broth (MERLIN Diagnostika GmbH, Bremen, Germany). The isolates were grouped into distinct resistotypes based on the presence of phenotypic resistance to relevant antimicrobials [24]. Interpretation of the results was based on the recommendations of the European Committee on Antimicrobial Susceptibility Testing (EUCAST) relevant at the time of isolation, with the exception of TIG, where the U.S. Food and Drug Administration (FDA) breakpoint (resistance of MIC > 4 mg/L) was applied [25,26]. Intermediate results were grouped with and reported as resistant [22]. Classification of the isolates as (MDR; resistance to at least one agent in ≥3 antibiotic groups) was based on Magiorakos et al. [27]; in subsequent analyses, isolates were divided into non-MDR and MDR. A multiple-antibiotic-resistance (MAR) index—ranging between 0 and 1—was calculated by dividing the total number of detected resistance to antimicrobials for each isolate by the total number of tested antimicrobials [24].

### 2.4. Phenotypic Detection of Carbapenemase and Metallo-β-Lactamase Production

To establish carbapenemase production in the *A. baummanni* included in the study, the isolates were subjected to the modified Hodge (cloverleaf) test, as previously described [28]. In the assay, MER disks (10 µg; Oxoid, Basingstoke, UK) were utilized and *Escherichia coli* ATCC 25922 was used as an indicator organism. Metallo-β-lactamase (MBL) production was tested using the imipenem/EDTA combined disk test (CDT), as described previously [29]. In preparation to this assay, imipenem/EDTA disks were prepared by adding 750 μg of a sterile 0.5 M EDTA solution to a 10 μg imipenem disk, and then disks were dried in a 37 °C incubator. Disks containing EDTA alone served as a negative control. The isolate was considered positive for MBLs if the inhibition zone diameter of the imipenem/EDTA disk increased by ≥7 mm compared to the imipenem disk alone [28].

### 2.5. Phenotypic Detection of Bacterial Efflux Pumps Contributing to the MDR Phenotype

To establish the effects of the overexpression of resistance-nodulation-division (RND) efflux pumps on the susceptibility of tested antimicrobials, a phenylalanine-arginine β-naphthylamide (PAβN) plate-based assay was performed, as described by Salehi et al. [30]. During the experiments, the concentration of PAβN (Sigma-Aldrich, St. Louis, MO, USA)—a compound with well-known efflux pump inhibitory activity—was 40 µg/mL in the agar base, while CIP, GEN, and MER were selected as test antimicrobials; a four-fold decrease in the MICs (determined by E-tests; Liofilchem, Roseto degli Abruzzi, Italy) of the antimicrobials in the presence of PAβN, compared to the MIC values without the inhibitor, was considered as positivity for efflux pump overexpression [30].

### 2.6. Biofilm Formation Assay

The capacity of respective *A. baumannii* isolates to produce biofilms was assessed using a microtiter-plate-based method previously described by Ramos-Vivas et al. [31]. In brief, fresh *A. baumannii* cultures (grown on Luria-Bertani (LB) agar) were inoculated into 5 mL of Luria-Bertani (LB) broth and incubated overnight at 37 °C. The following day, 180 μL of LB broth and 20 μL of *A. baumannii* suspension (set at 10^6^ CFU/mL) were measured onto 96-well flat-bottomed microtiter plates, to a final volume of 200 µL, and incubated for 24 h at 37 °C. After the incubation period, the supernatants were discarded and the wells were washed three times using 200 µL of phosphate-buffered saline (PBS; pH at 7.2), to remove planktonic cells, which may interfere with the interpretation of the results. After washing, the wells were fixed with 250 μL of methanol (Sigma-Aldrich, St. Louis, MO, USA) for 10 min and stained with a 1.0% crystal violet (CV; Sigma-Aldrich, St. Louis, MO, USA) solution for 15 min. Subsequently, the CV solution was discarded, and the wells were washed three times with purified water, to remove excess stains. The contents of the wells were re-suspended in 250 μL of 33% V/V% glacial acetic acid (Sigma-Aldrich, St. Louis, MO, USA), and the absorbance at 570 nm (OD_570_) was measured using a microtiter plate reader. All experiments were carried out in triplicate. The OD_570_ values of the measurements were recorded as the mean ± standard deviation (SD). The interpretation of the results was carried out based on the recommendations of Stepanovic et al. [32], namely, a cut-off value of optical density (OD_c_) was calculated using the following formula: OD_c_ = average OD of the negative control + (3 × standard deviations of negative control). Subsequently, isolates were classified into the following categories, based on their OD_570_ measurements: strong biofilm producer (OD > 4 × ODc); medium biofilm producer (4 × ODc ≥ OD > 2 × ODc); weak biofilm producer (2 × ODc ≥ OD > ODc); and nonbiofilm producer (OD ≤ ODc) [32].

### 2.7. Statistical Analysis

Descriptive statistical analysis (including means with ranges and percentages to characterize data) was performed using Microsoft Excel 2013 (Redmond, WA, USA, Microsoft Corp.). The normality of variables was tested using the Kolmogorov–Smirnov test. Independent sample *t*-tests were performed to compare measurements of OD_570_ (for biofilm production) between susceptible/resistant isolates to individual antibiotics, and among MDR and non-MDR *A. baumannii* isolates, respectively. Comparisons of groups (OD_570_ values for isolates resistant to respective antibiotics) were made by one-way analysis of variance (ANOVA) with Tukey’s post hoc tests. Statistical analyses were performed with SPSS software version 22 (IBM Corp., New York, NY, USA).

### 2.8. Ethical Considerations

The study was conducted in accordance with the Declaration of Helsinki and national and institutional ethical standards. Ethical approval for the study protocol was obtained from the Human Institutional and Regional Biomedical Research Ethics Committee, University of Szeged (registration number: 140/2021-SZTE [5019]).

## 3. Results

### 3.1. Antibiotic Susceptibility of Isolates Included in the Study

After re-identification with MALDI-TOF MS, all three hundred and nine (n = 309) isolates were verified as *A. baumannii*, and thus, they were included in the subsequent experiments. The antimicrobial resistance levels of the *A. baumannii* isolates included in the study were the following (in decreasing order): CIP 71.19% (n = 220), LEV 68.61% (n = 212), SXT 66.02% (n = 209), GEN 60.84% (n = 188), IMI 52.75% (n = 163), MER 51.46% (n = 159), AMI 38.51% (n = 119), TIG 38.51% (n = 44; MIC > 4 mg/L), and COL 2.27% (n = 7; MIC > 2 mg/L); overall, 42.72% (n = 132) of the isolates were classified as MDR, based on the criteria of Magiorakos et al. The distribution of the various resistotypes detected among *A. baumannii* isolates is presented in Table 1: twenty-one (I-XXI) different resistotypes were identified, with the most numerous being resistotype XVIII (resistant to CIP, LEV, IMI, MER, GEN, AMI, and SXT; 15.21%), XIV (resistant to CIP, LEV, GEN, AMI, and SXT; 9.40%), and IV (resistant to GEN and SXT; 9.06%).

### 3.2. Detection of Carbapenemase-Production and Efflux Pump-Overexpression Using Phenotypic Methods

To identify the contribution of various resistance mechanisms in the drug resistance of the relevant *A. baumannii* isolates, phenotypic tests were utilized. The modified Hodge test (MHT) was used to detect for the production of carbapenemases: overall, 22.65% (n = 70) of tested isolates (i.e., 42.94% of isolates resistant to either IMI, MER, or both) were positive for the phenotypic detection of carbapenemases in the MHT assay. MBL production was observed in 9.06% (n = 28) of the tested isolates (i.e., 17.18% of isolates resistant to either IMI, MER, or both) using the imipenem/EDTA combined disk test (CDT). Overexpression of the RND efflux pumps (based on the results of the PaβN screening agar) had significant effects on the MICs of MER in 14.24% (n = 44; i.e., 27.67% of isolates resistant to MER), while this was 6.05% in the case of GEN (n = 19; i.e., 10.11% of isolates resistant to GEN) and 27.51% in the case of CIP (n = 85; i.e., 38.64% of isolates resistant to CIP), which was demonstrated by the four-fold decrease in MICs detected in the presence of the efflux inhibitor compound. In the case of n = 17 and n = 8 isolates, simultaneous positivity of the efflux pump-overexpression-MHT test and efflux pump-overexpression-CDT test was detected, respectively.

### 3.3. Biofilm Formation among MDR and Non-MDR A. baumannii Isolates

**Figure 1:**
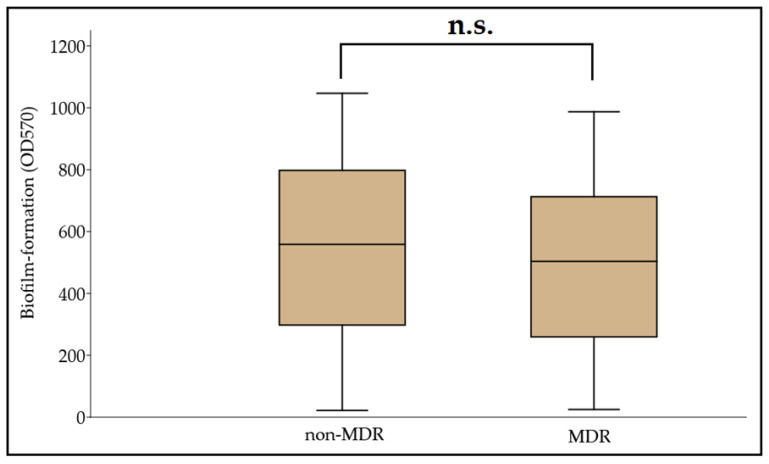
Comparison of biofilm-forming capacity among non-MDR and MDR *A. baumannii* isolates; n.s.: not significant (*p* = 0.072).

Biofilm formation assays were carried out in a microtiter plate-based platform, using CV staining. The OD_570_ values for the negative control (clinical isolate no. 59738) and the positive control (ATCC 19606) were 0.091 ± 0.008 and 0.388 ± 0.051, respectively. Therefore, the Odc value was set at 0.115, and the classification breakpoints were the following: non-biofilm producer: OD ≤ 0.115, weak biofilm producer: 0.230 ≥ OD > 0.115, medium biofilm producer: 0.460 ≥ OD > 0.230, and strong biofilm producer: OD > 0.460. Based on this, 9.39% (n = 29), 12.29% (n = 38), 22.97% (n = 71), and 55.35% (n = 170) were non-biofilm producers, and weak, moderate, and strong biofilm producers, respectively; OD_570_ values ranged between 0.022 ± 0.003 and 1.192 ± 0.086, with the median value being 0.501 (0.466, 0.534). In addition, 62.14% (n = 192) of isolates were more potent biofilm producers than the ATCC 19606 strain. A numerical but statistically not significant difference was identified between the MDR and non-MDR isolates regarding their biofilm-forming capacity overall (MDR: 0.495 ± 0.309 vs. non-MDR: 0.545 ± 0.283; *p* = 0.072) (Figure 1). During the analysis for individual associations between antibiotic resistance and biofilm propensity (where COL was excluded, due to the low number of resistant isolates), concordance was seen with the overall results, as no significant differences were shown for CIP (susceptible [S]: 0.556 ± 0.329 vs. resistant [R]: 0.499 ± 0.308; *p* = 0.55), LEV (S: 0.523 ± 0.299 vs. R: 0.508 ± 0.276; *p* = 0.63), SXT (S: 0.555 ± 0.262 vs. R: 0.492 ± 0.306; *p* = 0.28), GEN (S: 0.537 ± 0.329 vs. R: 0.463 ± 0.302; *p* = 0.66), AMI (S: 0.560 ± 0.283 vs. R: 0.481 ± 0.330; *p* = 0.99), TIG (S: 0.559 ± 0.349 vs. R: 0.471 ± 0.279; *p* = 0.81), IMI (S: 0.605 ± 0.283 vs. R: 0.538 ± 0.331; *p* = 0.63), and MER (S: 0.600 ± 0.315 vs. R: 0.538 ± 0.326; *p* = 0.68). While, based on numerical trends, MER-resistant isolates were the strongest biofilm producers, the ANOVA did not reveal significant differences (*p* = 0.067) between the biofilm production among antibiotic-resistant isolates.

## 4. Discussion

In recent years, *A. baumannii* infections have presented with alarming rates of MDR; however, the global spread of CR-AB was a critical hallmark of our fight against these pathogens, with their treatment emerging as an unmet medical need [33]. Carbapenem-resistance may be mediated by a multitude of mechanisms (often co-occurring in these isolates), including outer membrane impermeability (often due to deficiency or loss of porins), overexpression of bacterial efflux pump systems, modifications in the cell wall or penicillin-binding proteins (PBPs), and through the production of β-lactamases (hyperproduction of AmpC cephalosporinases and carbapenemases) hydrolyzing these drugs [34]. In *A. baumannii*, Ambler Class D (OXA-type carbapenemases: the species possesses an intrinsic β-lactamase gene (*bla*_OXA-51-like_), which is often used for the identification of this species), and Class B (metallo-β-lactamases; MBLs) are the most prevalent, while Class A (serine β-lactamases) enzymes have also been described on occasion [35]. The relevance of these enzymes has increased in recent years (either alone or co-existing with OXA-type enzymes) and represents an important therapeutic conundrum, as there are currently no licensed inhibitors for their inhibition in clinical use [36]. As there is limited clinical experience with novel, innovative β-lactam-type drugs (e.g., ceftazidime/avibactam, imipenem/relebactam, meropenem/vaborbactam, and cefiderocol) for *Acinetobacter* infections, non-β-lactam-type drugs often need to be administered [37]. For a number of years, colistin was the drug-of-choice for CR-AB infections, due to the fact that *Acinetobacter* spp. are still largely susceptible to this drug; however, resistance rates have been steadily emerging in the recent years [38]. Other potentially useful drugs include tigecycline (where susceptibility data allow for it) and novel tetracycline derivatives (eravacycline and omadacycline), plazomicin and delafloxacin, or high-dose combinations of existing drugs, such as meropenem-colistin [26,37]. Nevertheless, it must be noted that many of the above-listed antimicrobials may have debilitating adverse events and may not be suitable for a wide array of patients. Based on the findings of a systematic review, acquiring the MDR status in *Acinetobacter* spp. is most often due to resistance to aminoglycosides and carbapenems, while XDR and PDR (pan-drug resistance) are often associated with resistance to colistin, tigecycline, and other ancillary antibiotics [38]. As a consequence of the Severe Acute Respiratory Syndrome Coronavirus-2 (SARS-CoV-2) pandemic and the associated disease (COVID-19), patients undergoing mechanical ventilation have soared; these patients are particularly at risk for nosocomial *Acinetobacter* spp. infections, due to the introduction of assisted ventilation and the preceding viral infection, which predisposes patients to bacterial superinfections. Rapid increases are expected in the resistance rates of *Acinetobacter* spp. and other bacterial pathogens, associated with the extensive and prophylactic use of antimicrobials in relation with COVID-19 [39].

As a part of our study, a large pool (n = 309) of *A. baumannii* isolates from diverse geographical and clinical origins were subjected to phenotypic assays to characterize the possible correlation between biofilm-forming capacity and antimicrobial resistance in these isolates. In our pool of isolates, resistance rates were highest for the fluoroquinolones (~70%), while >42% were classified as MDR. Over 20% of isolates were positive for carbapenemase production (although our methods may not have enough sensitivity to reliably detect all carbapenemases relevant in *A. baumannii* [29]), ~9% were positive for MBL production, while in ~14%, carbapenem nonsusceptibility was affected by efflux pump overexpression (based on the phenotypic tests applied). The levels of MDR in the isolates were similar to the MDR rates in Central Europe; however, carbapenem-resistance rates were >50% [9]. Our results have highlighted that—based on susceptibility alone—colistin and tigecycline may still be optimal treatment choices for *A. baumannii*. A limitation of our study is that many additional antibiotics were not tested to establish the potential XDR or PDR status of the isolates [38].

The majority of isolates (62.14% overall) were strong biofilm producers, both in the MDR and non-MDR groups; our tests conducted did not verify a significant difference in biofilm formation based on the susceptibilities of the relevant isolates to the tested antimicrobials. Based on the reports from the recent literature, the rate of biofilm formation in *A. baumannii* is around 75–100%, which is similar to rates observed in NFGNB, and significantly higher than those of members of the Enterobacterales and Gram-positive bacteria (0–40%); additionally, many studies have shown that environmental *Acinetobacter* isolates often produce more robust biofilms—probably with the aim of being resistant to desiccation—compared to isolates from clinical origins [17,40]. Zeighami et al. reported on the virulence characteristics of one hundred *Acinetobacter* spp. isolated from ICU patients: 91% of isolates were XDR, while 32% were resistant to all antibiotics tested, and the presence of class I and class II integrons was revealed in 67% and 10%; all isolates (100%) were either moderate or strong biofilm producers, with 100%, 98%, 95%, 92%, and 81% with carriage of *csuE*, *pgaB*, *epsA*, *ptk*, *bfmS*, and the *ompA* biofilm-related genes, respectively [41]. The study of Hassan et al. involved n = 74 *A. baumannii* isolates (both from clinical material and from environmental origins, identified by the presence of the *bla*_OXA-51-like_ carbapenemase), which were assessed for the biofilm formation by three independent methods (CV tube method, CV tissue microtiter plates, and Congo Red Agar) in juxtaposition with their resistance. The majority of clinical isolates (>90%) were resistant to ciprofloxacin, gentamicin, and carbapenems, although no carbapenemase genes were found during molecular testing. In addition, 64.86% of isolates were classified as moderate and strong biofilm producers; biofilm formation was stronger in isolates susceptible to ciprofloxacin, gentamicin, and imipenem/meropenem [42]. Qi et al. included n = 272 *A. baumannii* isolates from various general hospitals in China, which had resistance rates to ciprofloxacin, ceftazidime, trimethoprim/sulfamethoxazole, imipenem, amikacin, and polymyxin B of 65.8%, 61.0%, 59.9%, 59.2%, 46.0%, and 3.7%, respectively. In addition, >72% classified as either MDR or XDR, and 23% were classified as strong biofilm producers, with non-MDR isolates producing a significantly more robust biofilm (this association was also verified for susceptibility to all individual antibiotics, apart from polymyxin B). Pulse-field gel electrophoresis (PFGE) analyses have revealed that the majority of nonsusceptible isolates—which were weak biofilm producers—belonged to the same eight main PFGE clusters [43].

Thummeepak et al. included n = 225 *A. baumannii* strains to perform phenotypic and molecular testing, during which 86.2% presented as MDR and 76.9% were biofilm producers; the presence of virulence and biofilm-related genes (*epsA*, *bap*, *ompA*, and *bfmS*) showed significant association with the MDR phenotype in these isolates; additionally, a higher number of biofilm producers were detected among gentamicin-resistant *A. baumannii* [44]. Greene et al. studied n = 145 clinical and environmental *A. baumannii* isolates for their biofilm-forming capacity: using cell death due to desiccation as an indicator, they found that non-MDR isolates were 2.7-times more susceptible to drying damage than the MDR isolates were [45]. Using *A. baumannii* clinical isolates and the ATCC 17978 reference strain as model organisms, Mayer et al. identified that operating quorum sensing (QS) systems are needed for functional motility and biofilm formation in vitro; their study highlighted that extracellular DNA is critical for the integrity of robust biofilms, in addition to showing that biofilm formation did not correlate with the antibiotic resistance of said isolates [46]. In addition, Selasi et al. noted that biofilm formation positively correlated with the expression level of many relevant virulence factors, such as surface proteins, pili, and the production of poly-β-(1-6)-N-acetylglucosamine (PNAG) and acyl-homoserine lactone (AHL) signal molecules [47].

Several authors have aimed to propose a definite genetic link between the propensity to form biofilms and antimicrobial resistance in *A. baumannii* [17]. In the study of Aziz et al., *A. baumannii* isolates—which carried the extended spectrum β-lactamase *bla*_PER-1_—produced a significantly more robust biofilm than the gene-deficient bacteria; their results showed that *bla*_PER-1_-positive isolates more efficiently adhered to epithelial cells in in vitro settings, and this attribute may be relevant in the early stages of biofilm production [48]. On the other hand, Gallant et al. showed contrasting findings for *P. aeruginosa*, where isolates presenting with and expressing the β-lactamase gene *bla*_TEM-1_ had limited ability to form biofilms, which was suggested to be a consequence of the low adhesive potential of these strains [49]. The biofilm-associated protein (Bap) is a cell surface protein in *Acinetobacter* spp.—homologous with the Bap protein found in *Staphylococcus* spp.—which has important roles to surface attachment and biofilm maturation. In the context of antibiotic resistance, it has been suggested that interactions of Bap and the outer membrane protein OmpA, which functions as the major porin *A. baumannii*, may have relevance in biofilm-forming capacity; namely, porin-deficient strains may show less pronounced biofilm production [18]. Our study emphasizes the need for additional experiments to assess the role biofilms have in the pathogenesis of *A. baumannii* infections.

## Figures and Tables

**Table 1:** Resistotype distribution and MAR indices of respective isolates.

Resistotypes	Resistance Pattern	MAR Index	MDR Status *	Ratio of Isolates (n, %)
I	*CIP*	0.111	non-MDR (57.28%; n = 177)	9 (2.91%)
II	*LEV*	0.111		10 (3.24%)
III	*SXT*	0.111		20 (6.47%)
IV	*GEN*, *SXT*	0.222		28 (9.06%)
V	*CIP*, *SXT*	0.222		3 (0.97%)
VI	*CIP*, *LEV*	0.222		14 (4.53%)
VII	*IMI*, *MER*, *SXT*	0.333		12 (3.88%)
VIII	*IMI*, *MER*, *GEN*	0.333		18 (5.83%)
IX	*CIP*, *LEV*, *SXT*	0.333		18 (5.83%)
X	*CIP*, *LEV*, *MER*	0.333		6 (1.94%)
XI	*CIP*, *LEV*, *IMI*	0.333		10 (3.24%)
XII	*CIP*, *LEV*, *GEN*	0.333		9 (2.91%)
XIII	*CIP*, *LEV*, *IMI*, *MER*	0.444		20 (6.47%)
XIV	*CIP*, *LEV*, *GEN*, *AMI*, *SXT*	0.555	MDR (42.72%; n = 132)	29 (9.40%)
XV	*CIP*, *LEV*, *GEN*, *AMI*, *SXT*, *TIG*	0.666		20 (6.47%)
XVI	*CIP*, *LEV*, *IMI*, *MER*, *GEN*, *SXT*	0.666		12 (3.88%)
XVII	*CIP*, *LEV*, *IMI*, *MER*, *GEN*, *TIG*	0.666		10 (3.24%)
XVIII	*CIP*, *LEV*, *IMI*, *MER*, *GEN*, *AMI*, *SXT*	0.777		47 (15.21%)
XIX	*CIP*, *LEV*, *IMI*, *MER*, *GEN*, *AMI*, *SXT*, *TIG*	0.888		7 (2.27%)
XX	*CIP*, *LEV*, *IMI*, *MER*, *GEN*, *AMI*, *SXT*, *COL*	0.888		5 (1.62%)
XXI	*CIP*, *LEV*, *IMI*, *MER*, *GEN*, *AMI*, *SXT*, *TIG*, *COL*	1.000		2 (0.65%)

* based on the criteria of Magiorakos et al. [27].

## Data Availability

All data generated during the study are presented in this paper.
